# Enhanced therapeutic efficacy of platinum-doxorubicin nanoparticles on colon and breast cancer cell lines

**DOI:** 10.1007/s00210-025-04080-4

**Published:** 2025-04-29

**Authors:** Ferdane Danışman Kalındemirtaş, Gökçe Erdemir Cilasun, Afşin Kariper

**Affiliations:** 1 Department of Physiology, Faculty of Medicine, Erzincan Binali Yıldırım University, Erzincan, Turkey; 2 Department of Medical Biology, Faculty of Medicine, Biruni University, Istanbul, Turkey; 3 Department of Science Education, Education Faculty, Erciyes University, Kayseri, Turkey

**Keywords:** Platinum nanoparticles, Doxorubicin, Cancer treatment, Cytotoxicity

## Abstract

In this study, platinum nanoparticles (PtNPs) were synthesized and their potential to improve the efficacy of doxorubicin (DOX) in cancer treatment was investigated. H2PtCl6, LiAlH4, and trisodium citrate were used during the synthesis of PtNPs. They were characterized using dynamic light scattering (DLS), Fourier transform infrared spectroscopy (FTIR), and scanning transmission electron microscopy (STEM). The diameter of the PtNPs was measured to be 21.72 nm without DOX loading and approximately 212 nm after DOX loading (DOX-PtNPs). FTIR confirmed the binding of DOX to PtNPs. In addition, MTT assays showed that DOX-PtNPs have a stronger effect on MCF-7 and HCT116 cancer cells than free DOX, even at low doses. The IC_50_ value for MCF-7 cells treated with DOX was determined to be 4.81 µg/ml, while it was significantly lower for the DOX-PtNP group at 0.64 µg/ml. A similar trend was observed in HCT116 cells, where the IC_50_ value for DOX was 5.03 µg/ml, while for DOX-PtNPs it was 0.62 µg/ml. In summary, the activity of DOX in these cells was increased approximately eightfold by PtNPs. Moreover, DOX-PtNPs showed no significant cytotoxic effects on normal HUVEC cells at low doses. Moreover, DOX-PtNPs enhanced apoptotic activity in HCT116 cells without inducing drug resistance as demonstrated by Rho123 staining and annexin/PI analyses. The significance of this study lies in the pioneering use of DOX-PtNPs in colon cancer, the synthesis of smaller PtNPs, the eightfold increase in the efficacy of DOX, and the demonstration that DOX-PtNPs do not significantly increase drug resistance.

**Supplementary Information:**

The online version contains supplementary material available at 10.1007/s00210-025-04080-4.

## Introduction

Cancer, one of the leading causes of death worldwide, is a complex disease in which cells multiply uncontrollably and spread to secondary organs. Colorectal cancer (CRC) ranks third in incidence and second in mortality worldwide (Bray et al. 2024). Additionally, breast cancer is one of the most common types of cancer in women (Liyanage et al. 2019). Surgical intervention and radiotherapy are used as a treatment for CRC and breast cancer. Conventional treatments can cause serious side effects. Nanotechnology-based drug carriers can potentially reduce systemic toxicity while increasing efficacy by targeting chemotherapeutic agents. In instances where traditional therapeutic modalities prove ineffective, nanomedicine, a subject of considerable interest in recent years, emerges as a promising alternative (Liyanage et al. 2019).

Metallic nanoparticles (NPs) possess the potential to address issues associated with traditional chemotherapy. These NPs are documented to enhance cancer treatment through improved targeting, gene silencing, and drug delivery (Sharma et al. 2018). Recently, researchers have presented findings on the selectivity capacity of functionally modified platinum nanoparticles (PtNPs) in the treatment of cancer cells, using specific methods. They are also expected to improve radiotherapy as they can accumulate specifically on the tumor. Compared to other metal nanoparticles, PtNPs are known to have similar cellular biocompatibility to gold nanoparticles and higher catalytic activity than palladium nanoparticles (Shiny et al. 2014; Martins et al. 2017). However, as PtNPs have a stronger cytotoxic effect, platinum ions can interact with DNA and inhibit its replication and can be used in cancer therapy (Mohammadi et al. 2013; Hashimoto et al. 2016). These PtNPs have been shown to have anti-oxidant properties that can inhibit tumor progression, which is useful in combating resistant cancers. In addition, the exceptional physicochemical and biological properties of PtNPs make them suitable for diverse applications such as nanomedicine, radiotherapy, and nanocatalysts. Moreover, PtNPs can be synergistically combined with chemotherapy and radiotherapy to enhance the efficacy of cancer treatments while minimizing adverse effects. In summary, further development of PtNPs as anti-cancer agents holds significant potential for future biomedical applications involving disease detection, drug delivery, and multifunctional therapeutic approaches (Fu et al. 2021; Klebowski et al. 2022; Yerpude et al. 2023).

Doxorubicin (DOX) is a broad-spectrum anthracycline derivative that was first extracted from *Streptomyces peucetius* bacteria in Italy in the 1960s. The Food and Drug Administration (FDA) has recognized that DOX has a significant therapeutic effect and is one of the effective chemotherapy drugs for many cancer types such as breast, prostate, and thyroid (Carvalho et al. 2009). DOX suppresses the functions of DNA and RNA polymerase, interferes with DNA replication and RNA transcription, and prevents DNA repair by inhibiting topoisomerase II. It can drive cells to apoptosis via the extrinsic pathway by affecting caspase activity and via the intrinsic pathway due to the increase in the amount of intracellular reactive oxygen species (ROS) (Bukowski et al. 2020). Although widely used in chemotherapy, it is a highly cytotoxic substance for non-cancer cells, with the best known side effect being cardiotoxicity (Kciuk et al. 2023).

Conventional DOX delivery systems often provide limited targeting, which can lead to high systemic toxicity and poorer accumulation of the drug at tumor sites. Many existing delivery systems are not sufficiently selective for most cancer cells and can cause significant damage to healthy tissues (Aloss and Hamar 2023; Alneghery et al. 2024; Elbeltagi et al. 2025). DOX-conjugated PtNPs improve targeting efficiency by enhancing drug accumulation at tumor sites due to their desirable physicochemical properties such as size and surface properties, thus allowing the nanoparticles to exploit the enhanced permeability and retention (EPR) effect characteristic of tumors. Furthermore, an important limitation of conventional DOX therapy is the development of multidrug resistance (MDR) in cancer cells (Shen et al. 2008). DOX-loaded PtNPs can bypass MDR mechanisms by utilizing a nanocarrier system that reduces the interaction of the drug with efflux pumps. In addition, PtNPs may enhance the therapeutic efficacy of DOX even in resistant cancer cells by inducing apoptosis through alternative pathways.

Barabadi et al. (2024) discussed the functionalization of metal-based nanomaterials for cancer therapy, highlighting its role in improving biocompatibility, targeting, and therapeutic efficacy. These studies underscore the significance of nanoparticle surface functionalization in biomedical applications, particularly cancer treatment (Majeed et al. 2023; Barabadi et al. 2024). Metallic nanoparticles offer significant opportunities in nanomedicine, including drug delivery, imaging, and diagnostics. Surface functionalization enhances their biocompatibility and targeting capabilities, while their optical and magnetic properties enable innovative applications in cancer treatment and imaging. However, challenges such as biological toxicity and accumulation necessitate thorough toxicological and biocompatibility. In cancer treatment, specific peptides or biomolecules are added to their surface, enabling targeted drug delivery to cancer cells while minimizing damage to healthy tissues (Nayak et al. 2023; Barabadi et al. 2024). Majeed et al. (2023) used TAT-functionalized silver nanoparticles to induce apoptosis in breast adenocarcinoma cells, enhancing treatment specificity. Nanomaterial properties like shape, size, solubility, and surface chemistry affect biological interactions and outcomes surface charge and chemical functionality of different nanostructures can influence uptake, potentially leading to oxidative stress and DNA damage (Samadian et al. 2020; Elbeltagi et al. 2024; Alharbi et al. 2024). In view of the available information, there is an urgent need to minimize the adverse effects of chemotherapeutic agents used in cancer therapy. Therefore, the aim of our study was to investigate the potential of DOX bound to PtNPs to exert its therapeutic effect on cancer cells at a reduced dosage.

This study’s significance and novelty are underscored by the production of smaller PtNPs, the remarkable eightfold enhancement in the efficacy of DOX-loaded PtNPs against colon cancer cells, and compelling evidence that DOX-PtNPs do not significantly contribute to drug resistance. Notably, this is the first study to investigate DOX-PtNPs in colon cancer.

## Materials and methods

### Materials

The American Type Culture Collection (ATCC) provided the cell lines MCF-7 and HCT116. At the chemical level, Dulbecco’s Modified Eagle’s Medium (DMEM), dimethyl sulfoxide (DMSO), Roswell Park Memorial Institute (RPMI), and 3-(4,5-dimethylthiazol-2-yl)−2,5-diphenyltetrazolium bromide kit (MTT) were supplied by Sigma. These were used for cellular growth. Gibco (UK) supplied penicillin, streptomycin, and trypsin, while Gibco (USA) and Sigma (St. Louis, USA) provided the fetal bovine serum (FBS). Thermo Fisher (USA) provided the 20 × phosphate-buffered saline (PBS) used in the release experiments. The Annexin-V FITC kit was purchased from Beckman Coulter in the USA. The stock solution of 1000 mg/ml H_2_PtCl_6_ (in 2 M HCl water solution), purchased from Sigma-Aldrich, was of analytical purity. The other reagents, namely LiAlH_4_ and trisodium citrate, were also purchased from Sigma-Aldrich in analytical purity.

#### Synthesizing of PtNPs and DOX-PtNPs

The synthesis of nanoplatinum was achieved by utilizing a 1000 mg/ml H_2_PtCl_6_ standard solution as the source of platinum. This solution was diluted to 1 ml and added to 10 ml of distilled water, which was then stirred. Subsequently, 100 mg of solid LiAlH_4_ powder was added to the mixture in 20 mg portions. The temperature of the solution was then increased to 90 °C. The heating process was stopped when the color of the solution turned purple at 90 °C and it was understood that reduction had occurred. Then, stirring was continued until the mixture reached room temperature. The solution was then filtered with a 220 nm filter to remove any excess reducing and solid particles that may have been present in the mixture. Immediately afterward, the nanoPt (PtNPs) stock solution was mixed with DOX, and the carrier-drug nanocomplex (DOX-PtNPs) was prepared. The platinum concentration in the nanoPt solution was then diluted to 0.05 mg/ml. Conversely, a 2 mg/ml DOX stock solution was prepared and diluted to 0.04 mg/ml. The nanoplatinum and DOX solutions were mixed at appropriate concentrations for release and nanoencapsulation. The mixtures were sonicated in an ultrasonic bath for 5 min. The resulting DOX-PtNP complex was stored in a clean, light-free environment for analyses and experiments.

### Characterizing of DOX-PtNPs

#### DLS analysis of PtNPs and DOX-PtNPs

Particle size measurements were performed at 22–23 °C using a Zetasizer NanoZS instrument, equipped with a 4 mW He–Ne laser at a wavelength of 633 nm and with detection occurring at an angle of 173°.

#### EDX-STEM analysis of PtNPs DOX-PtNP samples

The NanoPt and NanoPt/DOX samples were deposited onto a glass substrate and covered with Au/Pd, with a thickness of 450 nm, using a Polaron SC 7620 sputter coater. Subsequently, the samples were analyzed using field-emission scanning transmission electron microscopy (STEM) and energy-dispersive X-ray spectroscopy (EDX). To image liquid samples for analysis, the samples were deposited onto glass substrates, allowed to dry, and then placed on the instrument.

#### FTIR analysis of PtNPs and DOX-PtNPs

The Fourier transform ınfrared (FTIR) spectroscopic evaluation of the nanoparticles containing nanoplatinum (Pt) and doxorubicin-conjugated platinum nanoparticles (DOX-PtNPs) was carefully undertaken with the application of a sophisticated PerkinElmer spectrometer, noted for its superior precision and reliability in the characterization of molecular compositions through the measurement of infrared absorption spectra.

### Absorption measurements of PtNPs and DOX-PtNPs

The Hach Lange DR 5000 spectrophotometer was employed for the assessment of the absorbance of aqueous solutions comprising plastics. In the course of this assessment, water served as the reference for measurements conducted across a wavelength range of 200–1100 nm.

### Drug release studies

The dilution procedures for drug release were conducted in a phosphate-buffered saline (PBS) medium. The time-dependent release of the drug-carrier complex at the end of 6–12–24–48–72 h was then followed by FTIR analyses. For this purpose, the absorption change in the vibrational spectrum of a functional group belonging to the drug or the chemical bond forming the DOX-PtNP complex was analyzed.

**Fig. 1:**
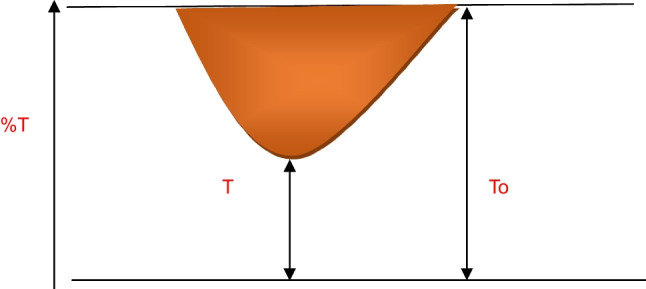
IR spectrum of a functional group

According to the Lambert–Beer law, the ratio of the intensity of the infrared absorption of the sample (I/Io) is equal to the molar absorptivity (*ε*) of the sample multiplied by the concentration of the sample (*C*) and the path length (*L*). This equation can also be applied to infrared spectroscopy. In general, the infrared spectrum of a prominent functional group in the structure is preferred, as shown in Fig. 1.

Figure 1 illustrates the specific vibration absorption peak of the sample. The relationship between the value of the maximum point of this peak and the value of the total absorption peak corresponds to the equation of Lambert–Beer law. The *C* concentration value can be easily calculated using the formula Abs = -log T = T/To = εLC. Consequently, the amount of drug incorporated into the solid nanocarrier will either decrease or increase the intensity of the absorption peak due to the binding of this functional group. This is because the intensity of the absorption peak decreases when other structures bind to the functional groups of the structure. This provides information about the concentration of this structure in the environment. The amount of substance used in direct release studies can be determined by working with the solution of the drug in pure form with a known initial concentration. Some of the added drug will bind to the nanomaterial, but some of the drug will not. The amount of unbound drug is measured and calculated (the amount of drug released that is not bound to the nanostructure). The reference is again pure water with soil correction (Erdik et al. 2001).

The cumulative release (%) of the drug was calculated as the following (Gohel et al. 2000; El-Kady et al. 2021):

$$Drug\;loading\;efficiency = {W}_{(total\;drug)}-{W}_{(free\;drug)} / {W}_{(total\;drug)} \times 100$$

### Nanoencapsulation

Nanoencapsulation is the process by which a drug is surrounded by a nanoparticle. It is a measure of the amount of drug that can attach to the nanoparticle. Some sources present this as a ratio, while others present it as a percentage ratio. Efficiency encapasilation (EE%) = amount of drug attached to the surface/total amount of drug × 100. In this experiment, the amount of drug was increased for a fixed amount of PtNPs: 1:1, 1:2, 1:3, 1:4, and 1:5. As in the previous oscillation experiment, the amount of drug attached to the PtNPs was determined by vibrational spectroscopy. The ability of the nanocarrier to transport the drug to the desired region is crucial for the success of this process (Dupeyrón et al. 2013).

### Cell culture studies

The widely used MTT test was applied to determine the difference in effect between the prepared nanodrug form and the chemotherapy drug currently in use. In this context, MCF-7 was selected as breast cancer cell line, HCT116 as colon cancer cell line, and HUVEC as a control healthy cell line. Cells were cultured in Dulbecco’s Modified Eagle Medium (DMEM, Gibco) containing 10% fetal bovine serum (FBS, Gibco) and 1% penicillin/streptomycin in a humidified air incubator with 5% CO_2_ maintained at 37 °C. When the cell density in the flask reached 80%, the flask was washed with PBS and 0.25% Trypsin–EDTA was applied to separate the cells from the flask bottom. This process, called passaging, was continued until sufficient cell numbers were obtained. When a sufficient cell number was reached, the cells were again removed from the flask bottom with 0.25% Trypsin–EDTA, and the enzyme was neutralized using a complete culture medium containing serum. After necessary centrifugation and cell counting, the cells were used in MTT assays. In this study, HUVEC cells were used as control cells in comparison with cancer cells. In addition, untreated group was used as control for each cell separately and the effect of DOX-PtNPs in each cell group was evaluated by comparing with free DOX.

#### MTT assay

The MTT assay is a frequently used rapid, reliable, and quantitative method that can determine the number of viable cells based on cell metabolic activity. MTT (3-(4,5-dimethylthiazol-2-yl)−2,5-diphenyltetrazolium bromide) is a water-soluble tetrazolium salt. Cell viability can be determined by the conversion of the tetrazolium salt into formazan crystals by the mitochondrial NADH/NADPH-dependent dehydrogenase activity in proliferating cells (Mosmann 1983).

Concentration ranges of 0.2–1–2.5–5 µg/ml were used for both DOX and nanoDOX drug forms (DOX-PtNPs). MTT assays were performed in 96-well plates. Cells were seeded with 10^4^ cells in each well. The determined drug concentrations were applied with at least 3 replicates of each concentration. Cells were incubated for 48 h at 37 °C in a humidified air incubator with 5% CO_2_. After 72 h, the supernatants in the wells were removed and 10 μl of MTT (5 mg/ml-Sigma) solution was added to each well. The wells were then kept at 37 °C in a humidified atmosphere with 5% CO_2_ in the dark for 4 h. During this time, MTT taken up by living cells was transformed into water-insoluble purple formazan crystals (Mosmann 1983). These formazan crystals were dissolved in 100 μl of dimethyl sulfoxide (DMSO) per well and the optical density of the solution was measured using a microplate spectrophotometer at a wavelength of 570 nm. Graphs and statistical analysis of the obtained data were performed using GraphPad Prism software.

#### AnnexinV/PI assay

Annexin V/PI assay was performed to determine the type of cell death (apoptosis or necrosis) the cells in the control and drug groups underwent. For this method, the FITC Annexin V Apoptosis Detection Kit with PI (Biolegend) specially designed to identify apoptotic and necrotic cells was used. Annexin V is commonly used to detect apoptotic cells by its ability to bind to phosphatidylserine, a marker of apoptosis when it is in the outer leaflet of the plasma membrane. Propidium iodide is a fluorescent intercalating agent that can stain cells and nucleic acids and bind to DNA.

Based on the data determined by MTT assay, 5 µg/ml DOX and 5 µg/ml DOX-PtNPs were applied to determine apoptotic activity. In other words, the same amount of DOX was used in both groups to compare the rates of apoptosis in the DOX and DOX-PtNPs groups. The cells were incubated for 48 h at 37 °C in a humidified atmosphere with 5% CO_2_. After 48 h, according to the manufacturer’s instruction, the collected cells were suspended in a binding buffer and dispensed into tubes. Then, the cells were stained with Annexin V and PI and incubated in the dark for 15 min at room temperature. Finally, the binding buffer was added to each tube, and flow cytometry analysis was performed. In the analysis results, Annexin V + and PI + cells were considered as late apoptotic, Annexin V + and PI—cells as early apoptotic, and Annexin V—and PI + cells as necrotic.

#### Fluorescence staining

Based on the data obtained from MTT and Annexin V/PI assay studies, fluorescent staining was performed using HCT116 cells in which the nanodrug form was highly effective. Hoechst staining was performed to show the type of cell death with one staining method and rhodamine 123 staining was performed to show the drug resistance.

#### Hoechst staining

Hoechst 33,342 is a dye that can pass through the cell membrane and bind to DNA. It is used to stain the nuclei of all living and dead cells (Atale et al. 2014). For the Hoechst staining, cells were seeded with 8 × 10^3^ cells in each well. The calculated IC_90_ doses of the drugs were applied. Cells were incubated for 24 h at 37 °C in a humid atmosphere with 5% CO_2_. At the end of the time, the medium was removed and the cells were fixed with 3% volume fractionated glutaraldehyde. Each well was treated with 10 µl hoechst (1 µg/ml concentration Hoechst 33342, Sigma) and incubated for 15 min at room temperature. Then, they were subjected to gradual (30–100%) alcohol dehydration. Fluorescence images were taken at 385 nm using a Nikon microscope.

#### Rhodamine 123 staining

Rhodamine 123 (Rho123) mitochondrial membrane stain was prepared as in the fluorochrome hoechst staining method. A green fluorescent mitochondrial dye called rhodamine 123 stains mitochondria in live cells in a way that depends on membrane potential. Rho123 has been associated with drug resistance. Accordingly, if the cell stains, there is no drug resistance, but if it does not stain, there may be drug resistance (Millot et al. 1994a). For Rho123 staining, cells were seeded with 8 × 10^3^ cells in each well. The calculated IC_90_ doses of the drugs were applied. For 24 h, cells were incubated at 37 °C in a humidified atmosphere with 5% CO_2_. At the end of the time, the medium was removed and the cells were fixed with 3% volume fractionated glutaraldehyde. Five microliters of Rho123 (1 µg/ml concentration, Sigma) was applied to each well and incubated for 30 min at 37 °C in the dark. Then, they were subjected to gradual (30–100%) alcohol dehydration. Fluorescence images were taken at 470 nm using a Nikon microscope.

### Statistical analysis

Cell viability data were analyzed using GraphPad Prism 5.04 software. The Mann–Whitney test was used for statistical analysis of the data and to determine whether all doses of each group were significant compared to the control. *P* < 0.05 was considered statistically significant.

## Results and discussion

### Characterization results of DOX-PtNPs

A variety of nanocarriers, including polymer, quantum dots, chitosan, and metal-based nanocarriers, have been employed for biomedical applications. PtNPs, CQDs, CSNPs, and hydrogel-based nanocarriers exhibit highly effective nanoparticle properties and offer advantages in terms of biocompatibility and controlled drug release. All these nanoparticles contribute to targeted cancer therapy by enhancing drug delivery and increasing bioavailability and unique physicochemical properties. In our study, the particle size of unloaded PtNPs (21.72 nm) and DOX-loaded PtNPs (~ 212 nm) maintained the optimum range for cellular uptake, while in another study, smaller (< 10 nm) CQDs offered bioimaging properties and high dispersibility, but may have different drug loading capacity. CSNPs and hydrogel-based nanocarriers (~ 453.23 nm) provide controlled drug release but are relatively larger. The present study suggests that DOX-PtNPs may offer advantages in terms of efficient cellular internalization with drug loading efficiency and that DOX-PtNPs offer enhanced cytotoxic potential (Danışman-Kalındemirtaş et al. 2022; Kariper et al. 2022; Hossein Karami and Abdouss 2024; Karami et al. 2025a, b).

**Fig. 2:**
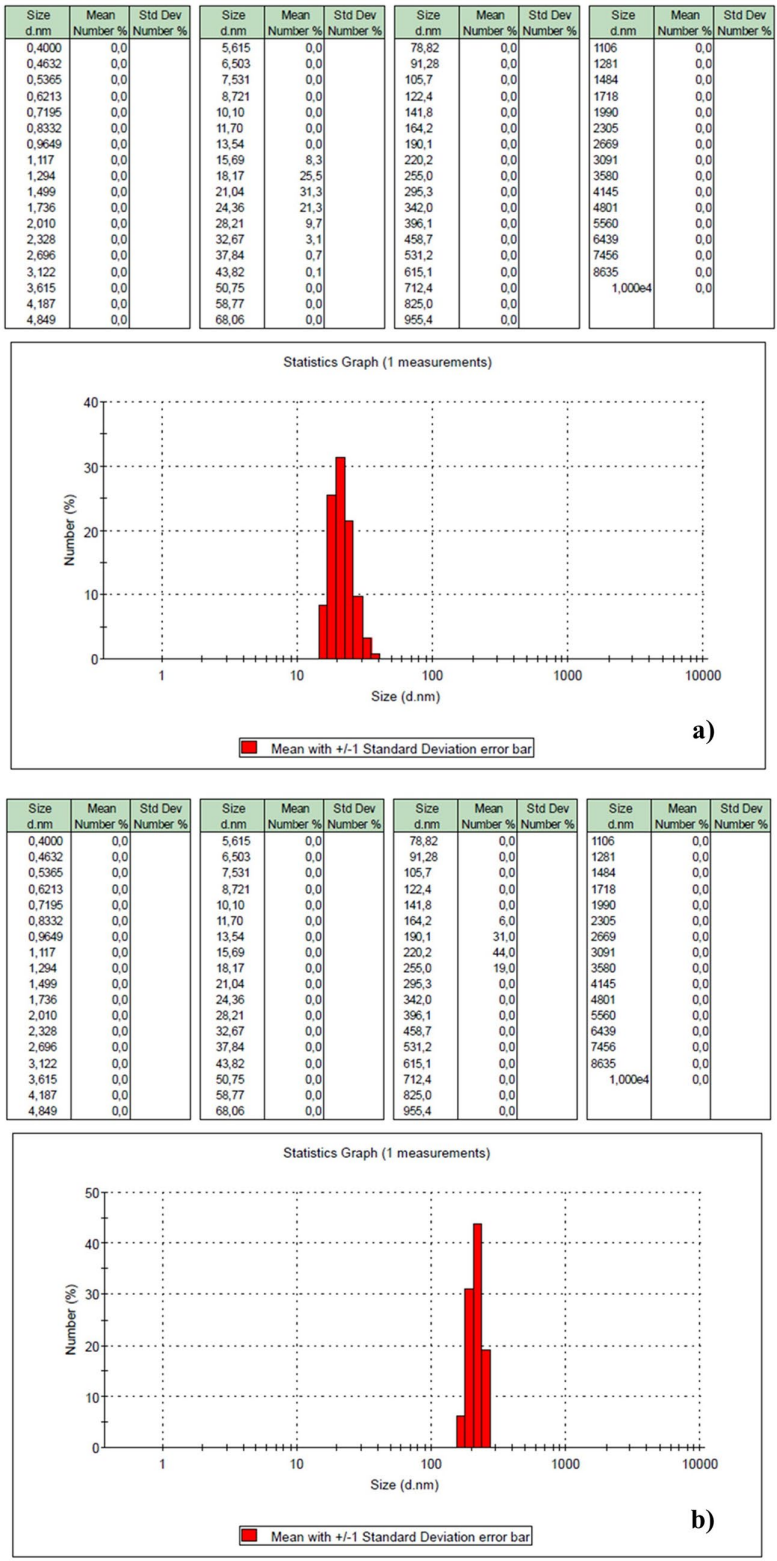

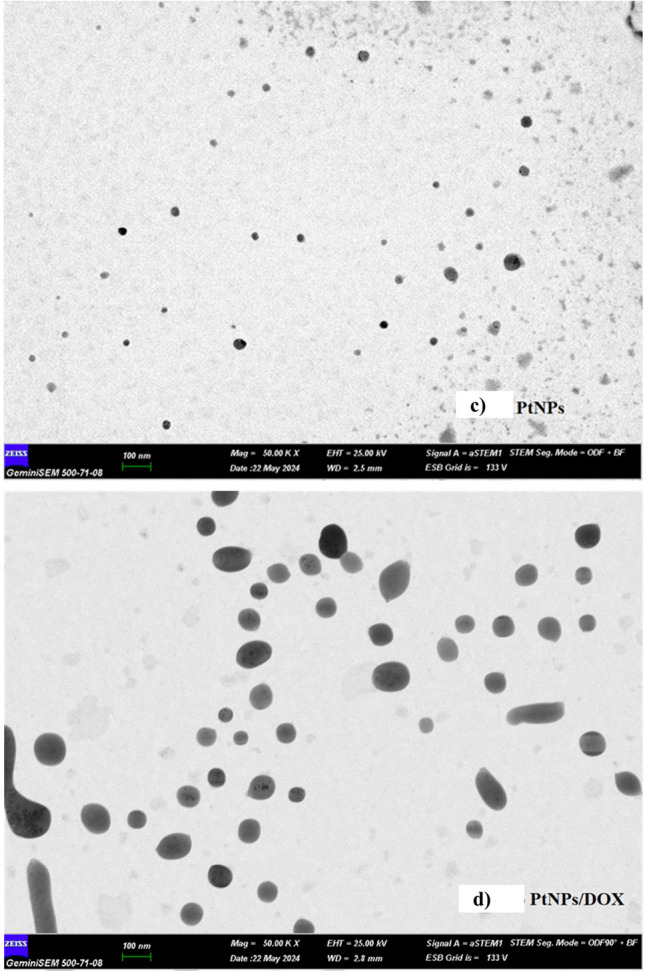

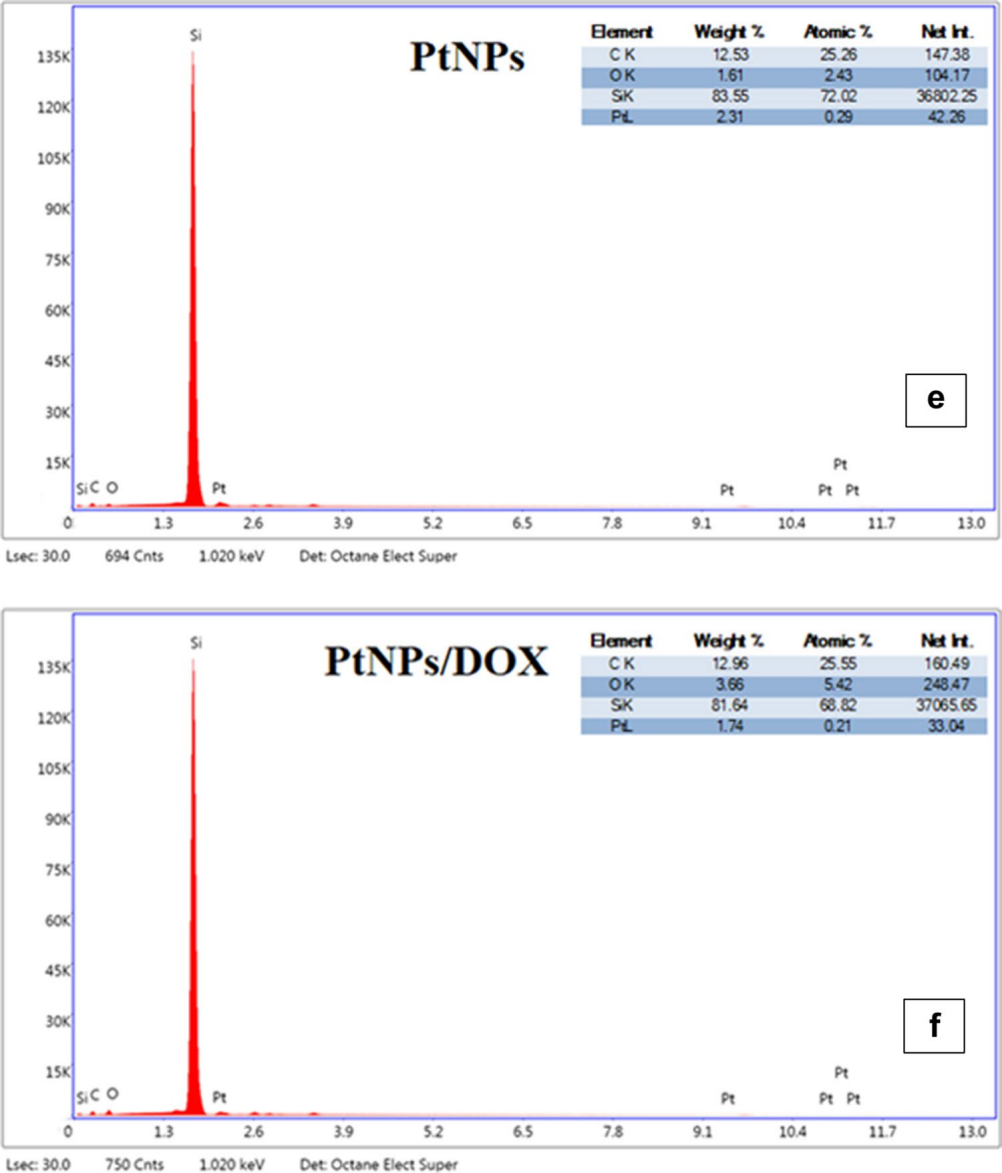
DLS analysis of the PtNPs (**a**) and DOX-PtNPs (**b**) STEM images of the PtNPs (**c**), DOX-PtNPs (**d**), and EDX analysis of the PtNPs (**e**) DOX-PtNPs (**f**)

As shown in Fig. 2a, PtNPs exhibited a particle size distribution ranging from 15 to 43 nm with an average particle size of approximately 21.72 nm (PDI, 0.476). In Fig. 2b, the particle size of the DOX-PtNPs complex exhibited an increase. The particle size ranged from 164 to 255 nm with an average particle size of approximately 212 nm (PDI, 0.652). This may be due to the physical or chemical adsorption of Pt^0^ to the electronegative oxygen atom (the Pauling electronegativity of oxygen is: *χ* = 3.44) in the hydroxyl groups in the DOX. When Pt^0^ is in the valence-free state (the Pauling electronegativity of platinum is: *χ* = 2.28), it is chemically and physically strongly attracted to electronegative atoms to satisfy its electron demand. Therefore, Pt^0^ tends to bond with oxygen because of the electron pairs available in oxygen. This significant increase in particle size is indicative of the successful binding of DOX to PtNPs. The size distribution of nanoparticles also gives an idea about their stability and distribution in biological environments. This also indicates that DOX-PtNPs have good stability and uniform distribution, which are essential for effective drug delivery. Thus, a more controlled and sustained release of the drug can be achieved, which helps in prolonged exposure of cancer cells to the therapeutic agent and controlled enhancement of the effect.

**Fig. 3:**
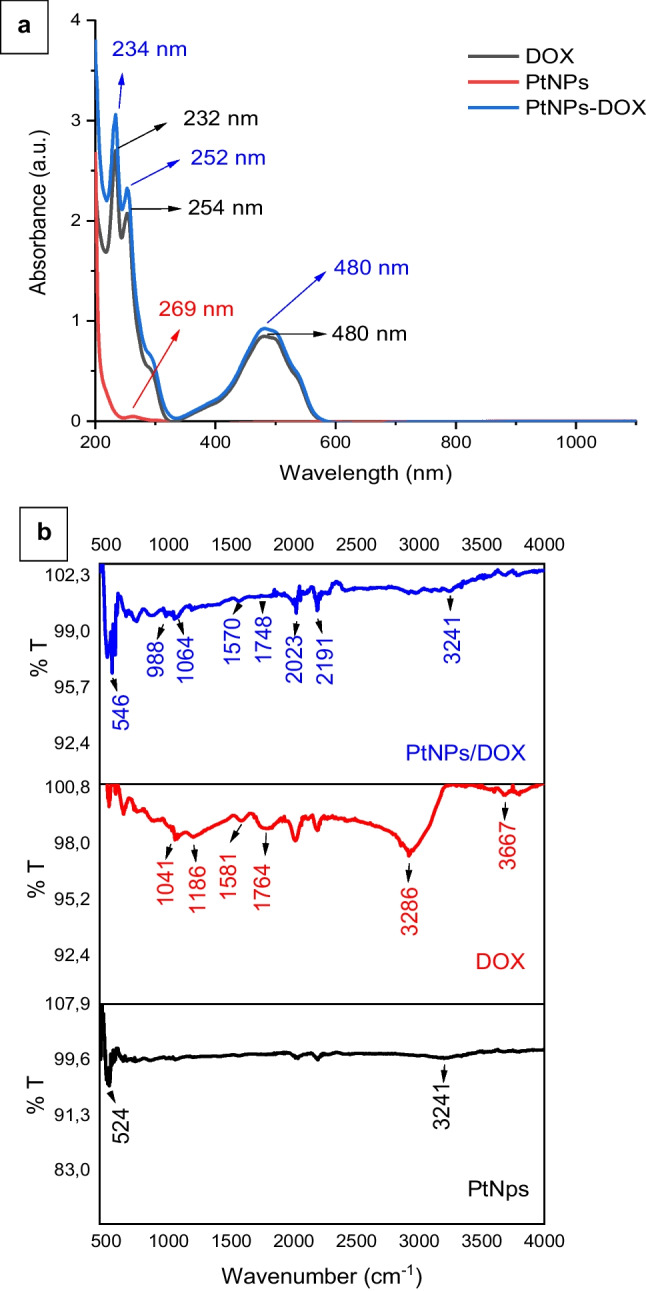
UV–VIS spectrum of the DOX, PtNPs, and PtNPs-DOX (**a**), FTIR spectrum of the PtNPs-DOX, DOX, and PtNPs (**b**)

The maximum absorbance values of DOX in different solvent environments vary. According to the available literature, the absorption of DOX in polar solvents is in the range of 378–550 nm (Fig. 3a) (Sikora et al. 2022). However, the maximum absorption wavelength is generally in the range of 450–500 nm. As the polarity interaction between solvent and solute increases, the maximum wavelength shifts to the red end of the spectrum. This is due to the jump of valence electrons in π bonds to the anti-bonding orbit, caused by electronegative atoms such as oxygen and nitrogen in the structure of DOX. Nanoplatin absorbs at maximum wavelengths of around 234 nm and 252 nm, depending on the changes in the synthesis method. It has been reported that the wavelength of nanoplatinum shifts to blue as the concentration increases and the particle size decreases as the access increases (Gharibshahi et al. 2017). DOX interacts with solvents in its environment, which influences the transitions of its valence electrons in π bonds. These transitions occur when electrons move from π-bonding orbitals to higher-energy anti-bonding π* orbitals. In polar solvents, strong interactions like hydrogen bonding or dipole–dipole forces stabilize the ground state and shift the absorption wavelength to the red region (bathochromic shift). DOX’s aromatic rings with extensive π-conjugation enhance its ability to absorb visible light. When DOX is conjugated to platinum nanoparticles (PtNPs), the interaction between DOX’s electronegative groups (like -OH and -NH₂) and PtNPs modifies the electronic environment, affecting the π → π* transition energies and the absorption spectrum (Gómez et al. 2023; Jachimska et al. 2024). This is because the absorption shifts to a lower wavelength with increasing particle size. Our findings are consistent with the results of DLS and the literature.

Figure 2c and d illustrate the scanning electron microscopy (STEM) analysis of the nanoparticles after they had been dried on a conductive layer. The particle size of PtNPs ranged from 20 to 40 nm, with some particles exceeding 50 nm, which aligns with the DLS results. When DOX was bound to PtNPs, the particle size increased, with some particles exceeding 100 nm and others ranging from 200 to 250 nm. However, in the dried samples prepared for STEM and EDX analysis, the particle sizes appeared smaller (100–150 nm) compared to DLS (164–255 nm) due to solvent evaporation during sample preparation, which caused the particles to attract water and appear slightly smaller. Despite this, the results are consistent with DLS findings. Figure 2e and f illustrate the findings of the EDX analyses conducted on the samples. As anticipated, the study revealed the presence of 0.29% platinum in the PtNPs, except for carbon originating from the base material. In the DOX-PtNP complex, the quantity of platinum diminished to 0.21% in conjunction with carbon and oxygen.

FTIR spectra of the samples dissolved in water are given in Fig. 3b and Table 1. Accordingly, in the FTIR spectrum of PtNPs, the vibration band of -OH groups at 3286 cm^−1^, [AlH_4_]- stretching vibration band at 1041 cm^−1^ (Ismail et al. 2014), and very weak ground vibration bands caused by CO_2_ at 2883 cm^−1^ and 2183 cm^−1^ were found. The presence of a vibration band at 1041 cm^−1^ confirms the successful reduction of platinum ions to form PtNPs. In the FTIR spectrum of DOX, the stretching vibration band of -NH groups at 3667 cm^−1^, stretching vibration band of -CH group at 2921 cm^−1^, stretch vibration bands of -CN group at 2183 and 2008 cm^−1^, stretch vibration bands of C = O group at 1764 cm^−1^, C = C stretch vibration bands of ring aromatic structure at 1581 cm^−1^, and stretch vibration bands of C–O–C group at 1041–1186 cm^−1^ were detected (Bansal et al. 2021). When DOX is bound to PtNPs, the vibration band of -OH groups at 3241 cm^−1^, stretch vibration bands of -CN group at 2191–2023 cm^−1^, and stretch vibration bands of C–O–C group at 1064–988 cm^−1^ were detected. In addition, vibration bands belonging to metal–oxygen (Pt-O) bond were detected at 501–546 cm^−1^ (Richardson et al. 1997). Amine groups at 3667 cm^−1^ are important for the interaction with PtNPs, as they can form hydrogen bonds or coordinate with the metal surface. Furthermore, the shifts in the FTIR peaks are evidence that DOX interacts with PtNPs via hydrogen bonding through -OH and -NH groups and possibly via coordination bonds through -CN and C = O groups. When DOX binds to PtNPs, the characteristic DOX peaks indicate that the drug retains its chemical structure after loading on PtNPs and are important in maintaining the therapeutic efficacy of DOX. The Pt-O stress vibrations also indicate that oxygen atoms in DOX interact with platinum atoms on the nanoparticle surface. This interaction will play an effective role in the controlled release of DOX from Pt nanoparticles.

**Table 1:** FTIR chart of the PtNPs-DOX, DOX, and PtNPs

Vibration mode	DOX (cm^−1^)	PtNPs (cm^−1^)	DOX-PtNPs (cm^−1^)	Interpretation
-OH stretching	3286	3241	3241	A shift in the hydroxyl (-OH) peak confirms hydrogen bonding between DOX and PtNPs
-NH stretching	3667	-	Disappeared	Indicates DOX binding to PtNPs via amine groups, likely due to electrostatic interactions
C = O (carbonyl)	1764	-	1748	A slight shift suggests the coordination of DOX’s carbonyl groups with platinum atoms
C = C (aromatic ring stretching)	1581	-	1570	A small shift suggests π-π interactions between DOX aromatic rings and PtNPs
C–O–C (ether groups)	1041–1186	1041	1064–988	Changes in this region suggest the potential bonding of ether groups with PtNPs
Pt-O (metal–oxygen bond)	-	524	501–546	Confirms the formation of Pt-O bonds, indicating strong chemical interaction between PtNPs and DOX

### Nanoencapsulation and drug release

**Fig. 4:**
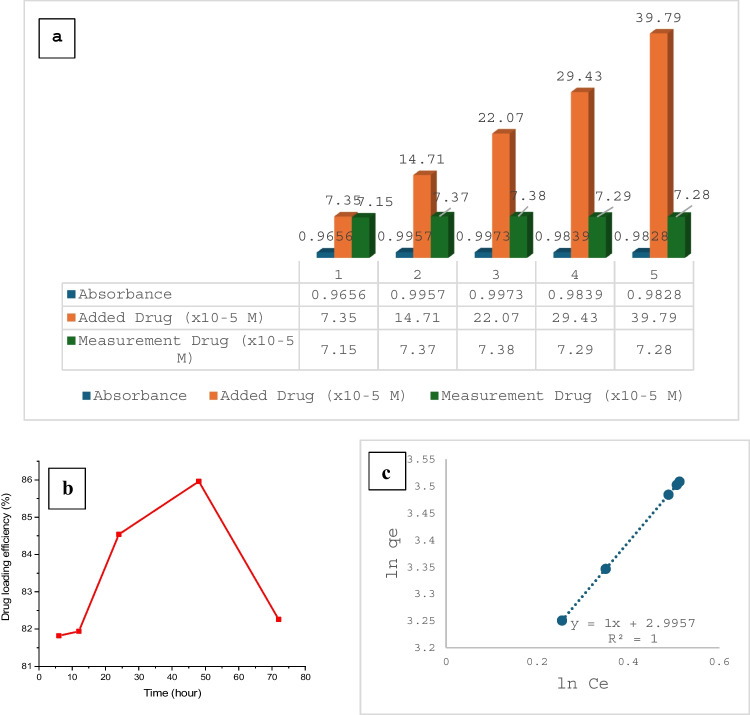
Amount of DOX adsorbed to PtNPs (**a**), drug loading efficiency via time (**b**), and Freundlich isotherm of DOX-PtNP nanocomplex (**c**)

To determine the maximum amount of nanoencapsulation, 0.04 mg/ml (7.35 × 10^−5^ M)—0.08 mg/ml (14.71 × 10^−5^ M)—0.12 mg/ml (22.07 × 10^−5^ M)—0.16 mg/ml (29.43 × 10^−5^ M)—0.20 mg/ml (39.79 × 10^−5^ M) were prepared by adding the appropriate amount of DOX to a 0.05 mg/ml nanoplatin solution. The amount of absorbed drug was then determined using the FTIR spectrum (see supplementary material, Fig. 1). The molar absorption coefficient of DOX is 13,500 L.mol/cm, as documented in the relevant literature (Nguyen et al. 2021). Absorption = -log T = T/To = εLC. Absorption values were read by taking the specific vibrational bands of platinum-oxygen groups at 548 cm^−1^ as reference. This is because PtNPs combine with the electronegative oxygen in this functional group to gain valence. The increase in absorption in this signal is also an indicator of the amount of doxorubicin bound to the carrier. Accordingly, the measured absorption, the amount of drug added to the system, and the amount of drug attached to the nanoplatin by absorption are given in Fig. 4a.

Figure 4a illustrates that PtNPs were maintained at a constant concentration of 12.80 × 10^−5^ M, approximately twice that of the drug, and retained approximately half of their concentration on the surface. The nanoencapsulation efficiency was found to be as high as 97.27%. However, when the concentration of PtNPs was increased to 2, 3, 4, or even 5 times that of the system, the nanoencapsulation efficiency decreased to 50.10%, 33.43%, 24.77%, and 18.29%, respectively. This indicates that nanoplatin is more effective in transporting the drug at half its concentration.

Drug release studies were performed in phosphate-buffered saline at 0, 2, 6, 12, 24, 48, and 72 h after loading 0.05 mg/ml DOX and 0.05 mg/ml nanoplatin. As described earlier, the release values were calculated using the absorption values obtained from the FTIR spectrum and the Lambert–Beer law. The most significant marker in the FTIR spectrum is the absorption of hydroxyl groups in DOX (3338 cm^−1^). The absorption of these functional groups is also an indicator of the quantity of drug released into the system. In addition to drug release, the adsorption isotherm of the drug-carrier system was also investigated. The relationship between the amount adsorbed by the unit adsorbent amount at constant temperature and equilibrium solution concentration is known as the adsorption isotherm. When the system attains balance during the adsorption procedure, the quantity of material adhered by a unit mass of adsorbent is a variable of temperature, concentration, pressure, or equilibrium pressure. The adsorption isotherm is also frequently used in drug release, and when the temperature is kept constant, this function is equivalent to the following equation:

The Freundlich isotherm is one of the most widely used isotherms in drug release.

$${q}_{e}={K}_{f}{C}_{e}^{1/n}$$

 

Kf = Freundlich constant *n*: constant (*n* > 1). If the logarithm of this equation is taken and linearized, the following equation is obtained;

$${lnq}_{e}={lnK}_{f}+\frac{1}{n}{lnC}_{e}$$

qe = adsorption capacity of adsorbent (mg/g, mol/g); Ce = concentration of adsorbate (adsorbed substance) at equilibrium (mg/l, mol/l); V = volume of solution (l); m = weight of adsorbent (g). Figure 4c shows the adsorption isotherm of the PtNPs-DOX complex (Haul 1982).

The Freundlich isotherm model is the most suitable model for heterogeneous adsorption systems such as the nanoparticle studies in this study. By binding drugs to nanoparticles, systems such as double-layer nanoencapsulation are formed. The Freundlich isotherm model is a method used to understand the adsorption character of systems with heterogeneous surface potential, such as multilayer adsorption.

The parameters “encapsulation efficiency” and “drug loading content” help to assess how effectively a drug is incorporated into a nanoparticle system and how much drug is loaded depending on the nanoparticle mass (Shen et al. 2017). Figure 4b shows the plot of drug loading efficiency (%) versus time for the DOX-PtNPs complex. According to Fig. 4b, the drug was released from the nanocomplex around 82% for 6–12 h. After 24–48 h, the release increased to 84–87%, and after 72 h the release decreased again. In other words, cumulatively, 82% of the drug was released in the first 6–12 h. 15.12% of the drug was released at 24 h. 2.50% of the drug was released at 48 h. 0.38% of the drug was released in the remaining 72 h. Figure 4c shows the plot of ${lnq}_{e}$ versus ${lnq}_{e}$ for Freundlich isotherm. Accordingly, the *n*-value is 1 and it can be seen that it has a high adsorption capacity. Kf value was 1.097. Freundlich isotherm expresses the degree of retention of a molecule on a surface in an adsorption process. According to the results of this study, the possibility of chemical adsorption on the PtNP surface is very high due to the Pt-oxygen interaction. However, if there is chemical interaction, the isothermal values are very high. Of course, one of the reasons why the amount of substance adsorbed on the surface remains at certain ratios is that a core–shell relationship is formed when a certain amount of the substance is chemically adsorbed on the surface. However, the formation of a third layer on top of this double layer is not expected. The isotherm results are also consistent with the nanoencapsulation results (97.27%) (Fig. 4c).

Tumors have a lower pH (~ 6.5) than normal tissues (pH 7.4) due to the Warburg effect, leading to lactic acid buildup. This pH difference enables pH-sensitive drug delivery systems, which remain stable at physiological pH but release drugs in the acidic tumor microenvironment, enhancing targeting and reducing systemic toxicity (Alsawaftah et al. 2022; Shinn et al. 2022).

The most appropriate parameter for DOX loading was selected as a concentration in the range of 0.04–0.20 mg/mL. These concentrations were found to be the best therapeutic doses for cancer cells and the lowest doses to minimize toxicity in normal cells. In addition, the highest loading efficiency of DOX on PtNPs was found to be 97.27%. This high efficiency indicates that the selected concentrations are appropriate and DOX binds to PtNPs efficiently.

### Cell culture

#### MTT assay

**Fig. 5:**
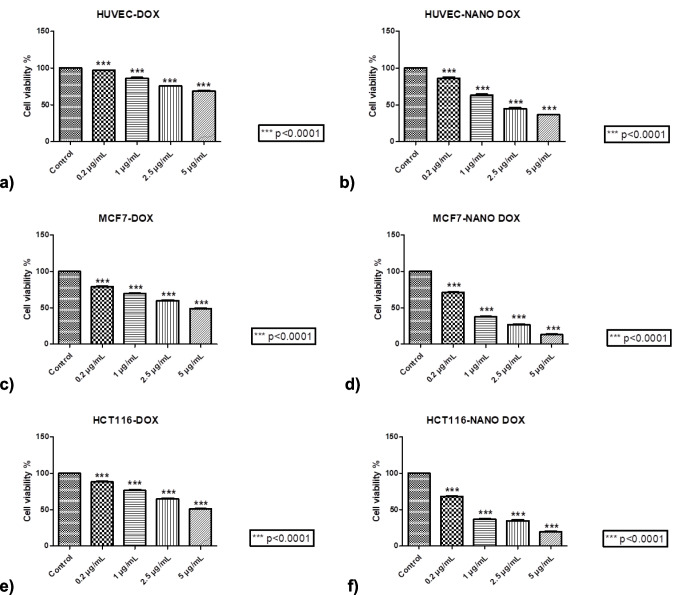
The cytotoxic effects of DOX and DOX-PtNPs at different concentrations on HUVEC, MCF-7, and HCT116 cells were evaluated by MTT assay. **a** DOX-treated HUVEC cells, **b** DOX-PtNP-treated HUVEC cells, **c** DOX-treated MCF-7 cells, **d** DOX-PtNP-treated MCF-7 cells, **e** DOX-treated HCT116 cells, and **f** DOX-PtNP-treated HCT116 cells. Concentrations are determined based on DOX. Data analysis of MTT experiments was performed using GraphPad Prism 5.04 application

The MTT assay results demonstrated that DOX-NPs exhibited greater efficacy than DOX against both MCF-7 (a breast cancer cell line) and HCT116 (a colon cancer cell line), even at low doses. In other words, DOX-NPs showed superior anti-cancer activity compared to DOX across both cell lines, with statistically significant findings. As shown in Table 2, the IC_50_ value for DOX-treated MCF-7 cells was 4.81 µg/ml, whereas for the DOX-PtNPs group, it was significantly lower at 0.64 µg/ml. Similarly, in HCT116 cells, the IC_50_ value for DOX was 5.03 µg/ml, while for DOX-PtNPs, it was 0.62 µg/ml (Fig. 5, Table 2). This indicates that PtNPs enhanced the efficacy of DOX by approximately eightfold in both cancer cell lines. Furthermore, the fact that normal HUVEC cells were not significantly affected at a DOX-PtNP concentration of 0.6 µg/ml, an effective dose for cancer cells, suggests that these nanoparticles selectively target cancer cells.

**Table 2:** The IC_50_ values (µg/ml) of DOX and DOX-PtNPs

Cell lines	The IC_50_ values (µg/ml) of DOX and DOX-PtNPs	
	DOX	DOX-PtNPs
HUVEC	> 5 ± 0.5	2.01 ± 0.3
MCF-7	4.81 ± 0.3	0.64 ± 0.1
HCT116	5.03 ± 0.6	0.62 ± 0.2

Each test was repeated at least three times. The results are presented as mean ± SD.

In a study on liver cancer, the anti-tumor effect of gold nanoparticles conjugated with conventional chemotherapy drugs cisplatin, DOX, and capecitabine was investigated. In vitro experiments showed that cancer cells treated with gold nanoparticles proliferated less than those treated with chemotherapy drugs alone. The result of the study supported that gold nanoparticles make cancer cells more sensitive to chemotherapy drugs (Tomuleasa et al. 2012). In another study, DOX encapsulated with AgNP, which is effective in the treatment of hepatocellular carcinoma, was investigated. The investigation involved 40 mice, which were divided into four groups. The results demonstrated that the DOX drug encapsulated with AgNP was more effective in reducing tumor growth and inducing apoptosis than the other groups (Said Shams El-Dine and Ali Abd El Kaream 2015). In a study investigating the anti-cancer effects of PtNPs produced by green synthesis, A549 cells, a lung cancer cell line, were studied. As a result of the experiments, it was determined that PtNPs alone could kill 47% of the cells at a dose of 160 µg/ml. Rokade et al. reported that PtNPs showed 49.65 ± 1.99% anti-cancer activity at a dose of 200 µg/ml, while PdNPs showed 36.26 ± 0.91% anti-cancer activity at the same dose (Rokade et al. 2018). Additionally, in another study, the anti-cancer activity of PtNPs has been demonstrated in vitro without the use of a chemotherapy drug (Baskaran et al. 2017; Yang et al. 2017; Şahin et al. 2018; Depciuch et al. 2020). In two separate studies utilizing MCF-7 breast cancer cells, the MTT assay was employed as a cell viability test, resulting in the identification of an IC_50_ value of 17.84 µg/ml in the study conducted by Sahin et al. and 31.2 µg/ml in the study by Baskaran et al. (Baskaran et al. 2017; Şahin et al. 2018). In another study with HCT116 human colon cancer cells, the IC_50_ value was found to be 20 µg/ml (Yang et al. 2017). When these studies are evaluated in conjunction, it can be concluded that PtNPs exert a notable anti-cancer effect on tumor cells, reducing their viability. Furthermore, our research findings are consistent with the conclusions of these studies. In a study investigating the effects of a doxorubicin-loaded chitosan-poloxamer in situ implant formulation for breast cancer treatment, it was determined that the formulation developed with an IC_50_ of 5 µg/ml of free DOX showed better cytotoxic activity in MCF-7 cell lines compared to free DOX (Sahoo et al. 2024). In a study aiming to use phospholipids from *Pseudomonas putida* bacteria as an abundant, safe, and accessible source to create nanoliposomes to deliver DOX to MCF-7 breast cancer cells, the anti-cancer effects of the produced nanocarrier were investigated by MTT assay. As a result of these experiments, the IC_50_ value of free DOX was 15 mg/ml, while that of the produced nanocarrier was 60 µg/ml (Asadi et al. 2024). The collective evidence from these studies demonstrates that nanoparticle-based interventions can mitigate the adverse effects of chemotherapy and anti-neoplastic agents by enhancing their therapeutic efficacy.

#### Apoptosis assay

**Fig. 6:**
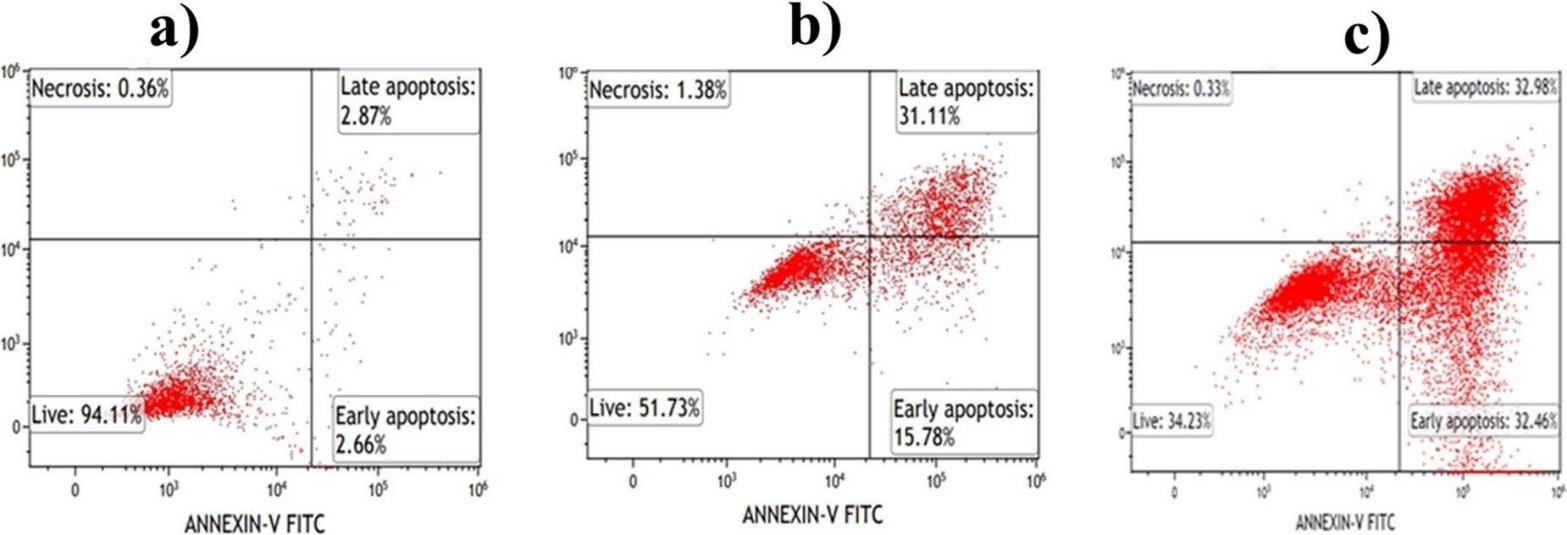
Apoptotic activity of DOX and DOX-PtNPs on HCT116 cells, control (untreated cells) (**a**), DOX (**b**), DOX-PtNPs (**c**)

In the subsequent phase of this study, HCT116 cells were employed to assess the apoptotic activity of the cell death pathway, with a comparison between the apoptotic activity of DOX and DOX-PtNPs. According to the results obtained from apoptosis assay by flow cytometry, DOX (5 µg/ml) killed 48.27% of the cells, which was determined to be apoptosis. It was observed that 15.78% of the cells underwent early apoptosis while 31.11% underwent late apoptosis. In addition, only 34.23% of the cells treated with DOX-PtNPs (5 µg/ml) remained viable. While 32.46% of the cells were in early apoptosis, 32.98% were in late apoptosis (Fig. 6, Table 3). Within the scope of these results, DOX-PtNPs were proven to be more effective than DOX by this method. These findings are in agreement with previous studies on the combination of DOX with various agents. For example, Khaleel et al. (2016) showed that the combination of DOX with didox and resveratrol reduced the IC_50_ value in HCT116 cells from 0.96 ± 0.02 to 0.4 ± 0.06 μM. This effect was attributed to increased expression of p53 and Bax, which promote apoptotic cell death (Khaleel et al. 2016). In another study, metformin and sodium oxamate compounds suppressed the PI3K/AKT signalling pathway in HCT116 cells and increased DOX-induced apoptosis, leading to cell cycle arrest and increased cell death (Coronel-Hernández et al. 2021).

**Table 3:** Apoptotic activity of DOX and DOX-PtNPs on HCT116 cells

	Live	Early apoptosis	Late apoptosis	Necrosis
Control	94.11	2.66	2.87	0.36
DOX	51.73	15.78	31.11	1.38
DOX-PtNPs	34.23	32.46	32.98	0.33

PtNPs can bind directly to guanine residues in DNA, forming intra-strand cross-links that block replication and transcription (Suchánková et al. 2012). In the literature, it is reported that apoptosis is induced by targeting Pt-based anti-cancer drugs, a platinum-based drug, to mitochondria, and only mitochondrial DNA (mtDNA) damage can trigger apoptosis without damaging nuclear DNA (Wisnovsky et al. 2013). Higher intracellular accumulation can be achieved by binding DOX to PtNPs, leading to mitochondrial depolarization and loss of mitochondrial membrane potential, which can induce apoptosis. In our study, it was observed that Pt-NPs induced apoptosis.

#### Fluorescence staınıng

##### Hoechst staining

Hoechst staining is a fluorescent method used to label DNA, assess nuclear morphology, identify apoptotic cells, and observe cell cycle changes (Atale et al. 2014). The data obtained from the MTT and apoptosis experiments were presented once more under a fluorescence microscope to facilitate further analysis. To this end, 24-h experiments were conducted by administering IC_90_ doses. The nuclear morphology of treated cells was compared to controls. Apoptotic cells typically appeared as condensed and fragmented nuclei. When cell density was analyzed, it was observed that the results were consistent with the MTT data. In addition, when the morphology of the cells was examined, the presence of pyknotic nuclei and DNA fragmentations was in parallel with the data of apoptosis experiments performed by flow cytometry.

##### Rhodamine 123 staining

**Fig. 7:**
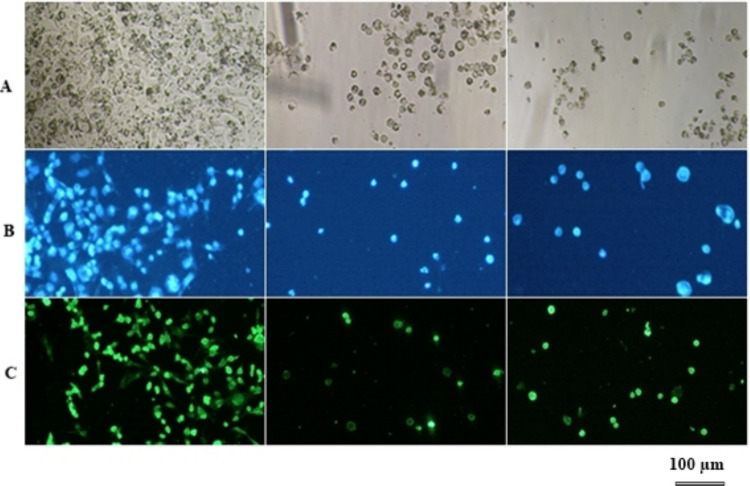
**A** Light microscopy images of untreated HCT116 control cells and after treatment with DOX and DOX-NPs. **B** Fluorescence microscope images of HCT116 cells after 24 h staining with Hoechst. **C** Fluorescence microscope images of HCT116 cells after 24 h staining with Rho123

According to the findings presented in Fig. 7b, the fluorochrome Hoechst staining method was shown to be a highly effective for the precise identification and analysis of apoptotic cells. Moreover, the presence of membrane bulges, which are indicators of the apoptotic process, decreased density in cells, and shrinkage in cells was observed after the application of treatments involving HCT116 cells, especially through the use of DOX or DOX-PtNP. Collectively, these findings can be seen as the effectiveness of the treatment associated with cellular apoptosis (Gomes et al. 2018). Rhodamine 123 (Rho123) staining was applied to HCT116 cells to determine the presence of possible drug resistance. Compared to the control, it was once again seen that the numerical density of the cells was consistent with the MTT assay data. The intracellular dye Rho123, which is dependent on the metabolic activity and differentiation potential of the cells, is maintained inside the cell at variable levels. This dye is removed from cells via the action of ABC transporter proteins, namely P-glycoprotein (P-gp). Rho123 is a useful tool for researching drug toxicity and the functional activity of P-gp in cultured cells since it is a substrate for the ABCB1/P-gp transporter (Millot et al. 1994b; Chekwube et al. 2020). Drug resistance observed in tumors can be explained by increased expression of carrier proteins in cancer cells that pump drugs out of the cell (Yehya et al. 2023). The fluorescence intensity of untreated cells was high, while the HCT116 cells treated with DOX had decreased fluorescence intensity (Fig. 7c). However, the group treated with DOX-PtNPs showed increased fluorescence intensity compared to DOX alone. These findings suggest that the DOX-treated cells may have higher drug resistance than the DOX-NP-treated cells. It is possible that the expression of ABC transporter proteins, such as P-gp, is higher in DOX-treated cells than in DOX-NP-treated cells. The study concludes that measuring the fluorescence intensity of Rho123 accumulation in cells can be a practical method for identifying drug-resistant cells and developing targeted therapies. Overall, DOX-PtNPs show the potential to reduce drug resistance.

Although platinum NPs have been synthesized before, DOX-loaded Pt-NPs are very limited and their effect on colorectal cancer cells was investigated in this study. Patel et al. produced Pt nanoparticles above 100 nm. They reported that Pt-DOX nanocomplex induced apoptosis in MCF-7 and MDA-MB-231 human breast cancer cells through high levels of reactive oxygen species production and DNA damage (Patel et al. 2021). Gurunathan et al. produced drug-unloaded Pt NPs above 30 nm and investigated the effect of DOX and Pt-NPs on osteosarcoma cells separately (Gurunathan et al. 2019). Our study demonstrates that the Pt-NPs produced (21 nm) are smaller than those in previous reports, while the Pt-DOX complex (212 nm) is an optimal size for enhanced cytotoxicity in colorectal cancer. The significant size increase upon DOX loading confirms effective drug incorporation, enabling controlled release. PtNPs boosted DOX cytotoxicity by approximately eightfold inducing multidrug resistance, as shown by Rho123 staining, a remarkable enhancement compared to prior studies. Unlike previous studies, this work specifically examined and confirmed their non-induction of MDR. The absence of toxicity in normal HUVEC cells suggests minimized side effects. These findings highlight DOX-PtNPs as a promising candidate for safe and effective cancer therapy.

The studies support that PtNPs increase the efficacy of chemotherapeutic agents and cancer cell death by inducing apoptosis and may render chemotherapy-resistant cells more sensitive. These findings suggest that DOX-PtNPs may be an effective approach to cancer treatment (Almarzoug et al. 2020). Additionally, PtNPs inhibit the DNA repair mechanisms of cancer cells by disrupting DNA repair, thereby increasing drug sensitivity (Nejdl et al. 2017). Thus, PtNPs may be more sensitive to chemotherapy-resistant cancer cells and provide a more effective treatment option. Kumari et al. (2016) reported that nanocarrier-bound drugs accumulate in cancer cells at higher concentrations, enhancing treatment efficacy (Kumari et al. 2016). Similarly, DOX-loaded PtNPs promote drug uptake and controlled release, potentially improving efficacy while reducing DOX-related side effects.

This study highlights the potential of DOX-NPs in enhancing chemotherapy efficacy and overcoming drug resistance. The results suggest that PtNPs improve treatment outcomes by inducing apoptosis, inhibiting DNA repair, and enhancing drug transport and release, offering a promising strategy for cancer therapy. The study’s main limitation is that DOX-PtNPs were evaluated exclusively in vitro. In vivo studies are important for further research to enhance comprehension of therapeutic effects.

## Conclusion

This study demonstrates the potential of PtNPs to improve the efficacy of DOX in the treatment of colorectal cancer. The results of this study suggest that DOX-PtNPs can selectively target cancer cells, reduce the necessary DOX dosage, and minimize toxicity to normal cells. The unique properties of PtNPs, including their small size, lage surface area, and exceptional biocompatibility, make them well-suited for multiple therapeutic applications. This research highlights the promising potential of nanomedicine in cancer therapy and paves the way for further investigations of PtNPs in clinical settings. Future studies should focus on in vivo testing and optimization of the drug delivery system to fully exploit the benefits of PtNPs in cancer treatment.

## Supplementary Information

Below is the link to the electronic supplementary material.

Supplementary file1 (DOCX 299 KB)

## Data Availability

All source data for this work (or generated in this study) are available upon reasonable request.
