# Supplementary material for: Enhanced therapeutic efficacy of platinum-doxorubicin nanoparticles on colon and breast cancer cell lines
Source: Naunyn Schmiedebergs Arch Pharmacol. 2025 Apr 29;398(10):14367–83. doi: 10.1007/s00210-025-04080-4 (PMC12511156; doi:10.1007/s00210-025-04080-4)
Supplement: Supplementary file 1 — Supplementary file1 (DOCX 299 KB) [file 210_2025_4080_MOESM1_ESM.docx]

**Supplementary Material**

“**Enhanced Therapeutic Efficacy of Platinum-Doxorubicin Nanoparticles on Colon and Breast Cancer Cell Lines**”

^a^Ferdane Danışman Kalındemirtaş, ^b^Gökçe Erdemir Cilasun, ^c^Afşin Kariper

^a^Erzincan Binali Yıldırım University, Faculty of Medicine, Department of Physiology, Erzincan, Turkey

^b^Biruni University, Faculty of Medicine, Department of Medical Biology, Istanbul, Turkey

^c^Erciyes University, Education Faculty, Department of Science Education, Kayseri, Turkey

**Supplementary Material Figure 1: FTIR spectrum of fixed amount of drug loaded on PtNPs.**

**Supplementary Material Figure 2 FTIR spectrum for drug release**
